# Comparative Evaluation of the Remineralization Potential of Monofluorophosphate, Casein Phosphopeptide-Amorphous Calcium Phosphate and Calcium Sodium Phosphosilicate on Demineralized Enamel Lesions: An In Vitro Study

**DOI:** 10.7759/cureus.3059

**Published:** 2018-07-27

**Authors:** Kavita Kumar, Sheela Sreedharan

**Affiliations:** 1 Pediatric Dentist, Innocent Smiles Dental Clinic, Ernakulam, IND; 2 Department of Pedodontics and Preventive Dentistry, Principal, Government Dental College, Alappuzha, IND

**Keywords:** remineralization, calcium sodium phosphosilicate, monofluorophosphate, confocal laser scanning microscopy, cpp-acp

## Abstract

Aim: The aim of the study is to compare the remineralization potential of monofluorophosphate, casein phosphopeptide-amorphous calcium phosphate (CPP-ACP), and calcium sodium phosphosilicate on demineralized enamel lesions.

Materials and methods: Enamel sections from 30 sound human premolar crowns were prepared and sectioned into quadrants. Early enamel lesions were created in each sample by immersion in a demineralizing solution for 72 hours. Of the four sections, the first quadrant (A) was not given any surface treatment, the second quadrant (B) was treated with monofluorophosphate dentifrice, the third (C) was treated with casein phosphopeptide-amorphous calcium phosphate (CPP-ACP), and the fourth (D) was treated with calcium sodium phosphosilicate while being subjected to a five-day pH cycling protocol. The sections were further cross-sectioned to expose the lesion depth and were then viewed under the confocal laser scanning microscope after staining with 0.1 mM rhodamine B dye for 24 hours. The two parameters evaluated were the cross-sectional demineralized lesion area and total fluorescence.

Results: Amongst the dentifrices tested, the lowest values for lesion area and total fluorescence were recorded by calcium sodium phosphosilicate (3874.1 µ^2^ and 107282.6, respectively), followed by casein phosphopeptide-amorphous calcium phosphate (5776.6 µ^2^ and 129470.8) and then by monofluorophosphate dentifrice (7371.2 µ^2^ and 233765.9) in increasing order. The highest values for lesion area and total fluorescence were recorded by the no treatment group (16449.2 µ^2^ and 759743.1). One-way analysis of variance (ANOVA) showed significant variations (p<0.01) between the groups and Scheffe multiple comparisons confirmed the significance (p<0.01) of intergroup variations.

Conclusion: The results of this study suggest that, among the three agents tested, calcium sodium phosphosilicate is the most effective remineralizing agent followed by casein phosphopeptide-amorphous calcium phosphate. Monofluorophosphate is the least effective remineralizing agent when tested under the conditions mentioned in this study.

## Introduction

Demineralization and remineralization occur in the mouth several times daily as a dynamic process, with the progression or reversal of dental caries being the end result. Whether a lesion will progress, stay the same, or reverse is determined by the balance between protective factors and pathological factors [1]. If the pathological factors that lead to demineralization predominate, then caries progresses. If the protective factors that lead to remineralization predominate, then caries is halted or reversed.

Though fluoride is the cornerstone of non-invasive caries management, its use predominantly leads to surface-only mineralization [2]. Surface-only remineralization does little to improve the aesthetics and structural properties of deeper lesions. Ideally, a remineralization system should supply stabilized bioavailable calcium, phosphate, and fluoride ions that favor subsurface mineral gain rather than deposition only in the surface layer.

The casein phosphopeptide-amorphous calcium phosphate technology has been developed based on the stabilizing properties of milk and salivary proteins. The casein phosphopeptides allow high concentrations of calcium, phosphate, and fluoride ions to be stabilized in solution, in a form that is bioavailable for the promotion of remineralization [3].

Calcium sodium phosphosilicate has been developed under the brand name Novamin (GlaxoSmithKline, Brentford, United Kingdom) and seems to be a promising alternative to promote the remineralization of early enamel lesions [4].

This in vitro study was, hence, carried out to compare the remineralization potential of monofluorophosphate, casein phosphopeptide-amorphous calcium phosphate, and calcium sodium phosphosilicate.

## Materials and methods

Collection and storage of teeth

Thirty maxillary first and second premolars extracted for orthodontic treatment with intact surfaces and no evidence of caries, hypoplasia, or restorations were used for the preparation of the specimens. All the teeth were stored in 0.1% thymol (w/v) solution until sterilization. They were cleaned of surface debris and were sterilized by autoclaving at 15 psi pressure at 121°C for 40 minutes [5].

Sectioning and mounting

**Figure 1:**
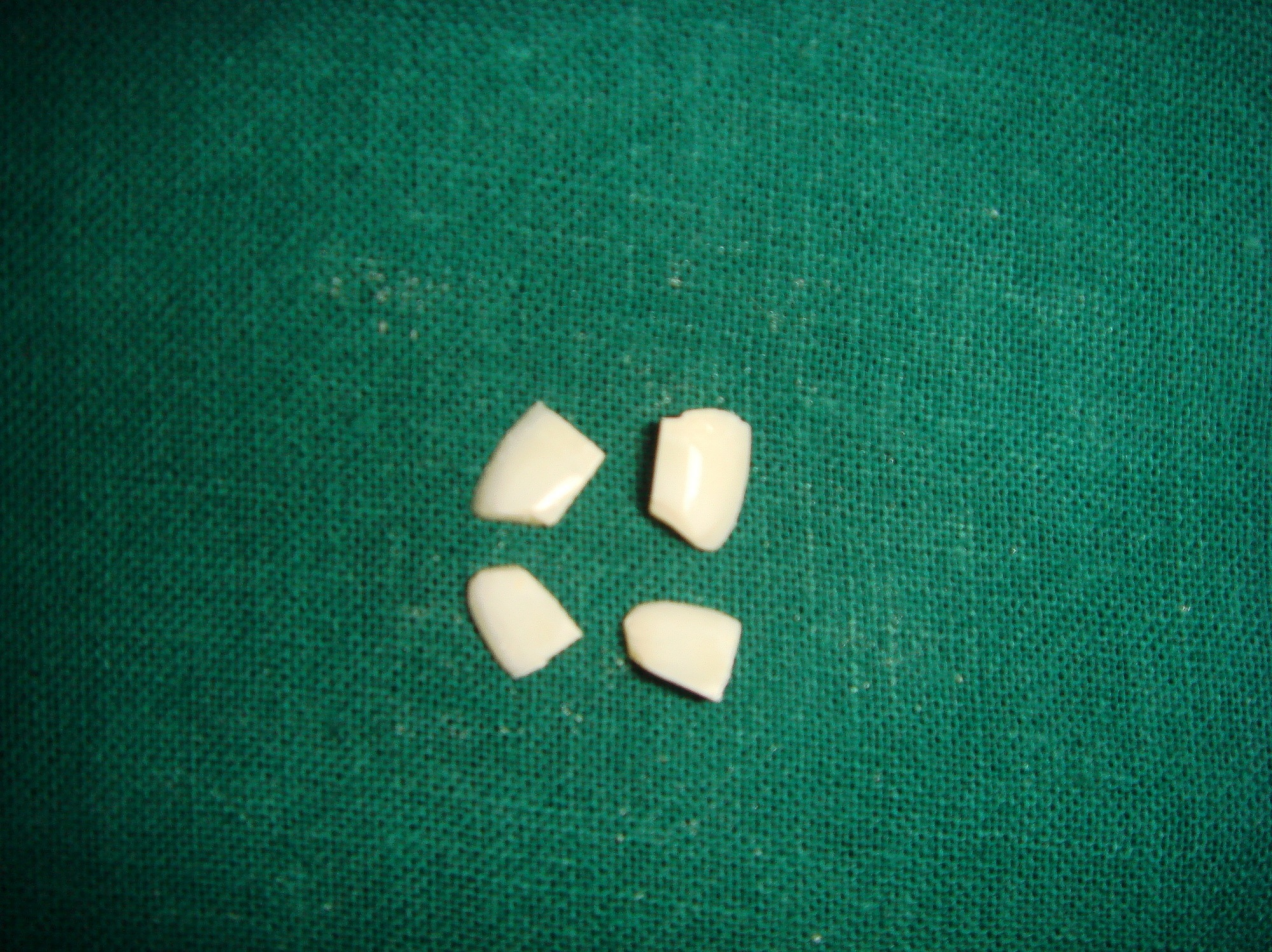
Sectioned quadrants of tooth

Each tooth was sectioned into quadrants along both mesiodistal and buccolingual planes and subjected to mechanical polishing with successive 120, 400, 600, and 1200 grit paper to expose the virgin enamel in the deeper layers [6] (Figure 1). 

**Figure 2:**
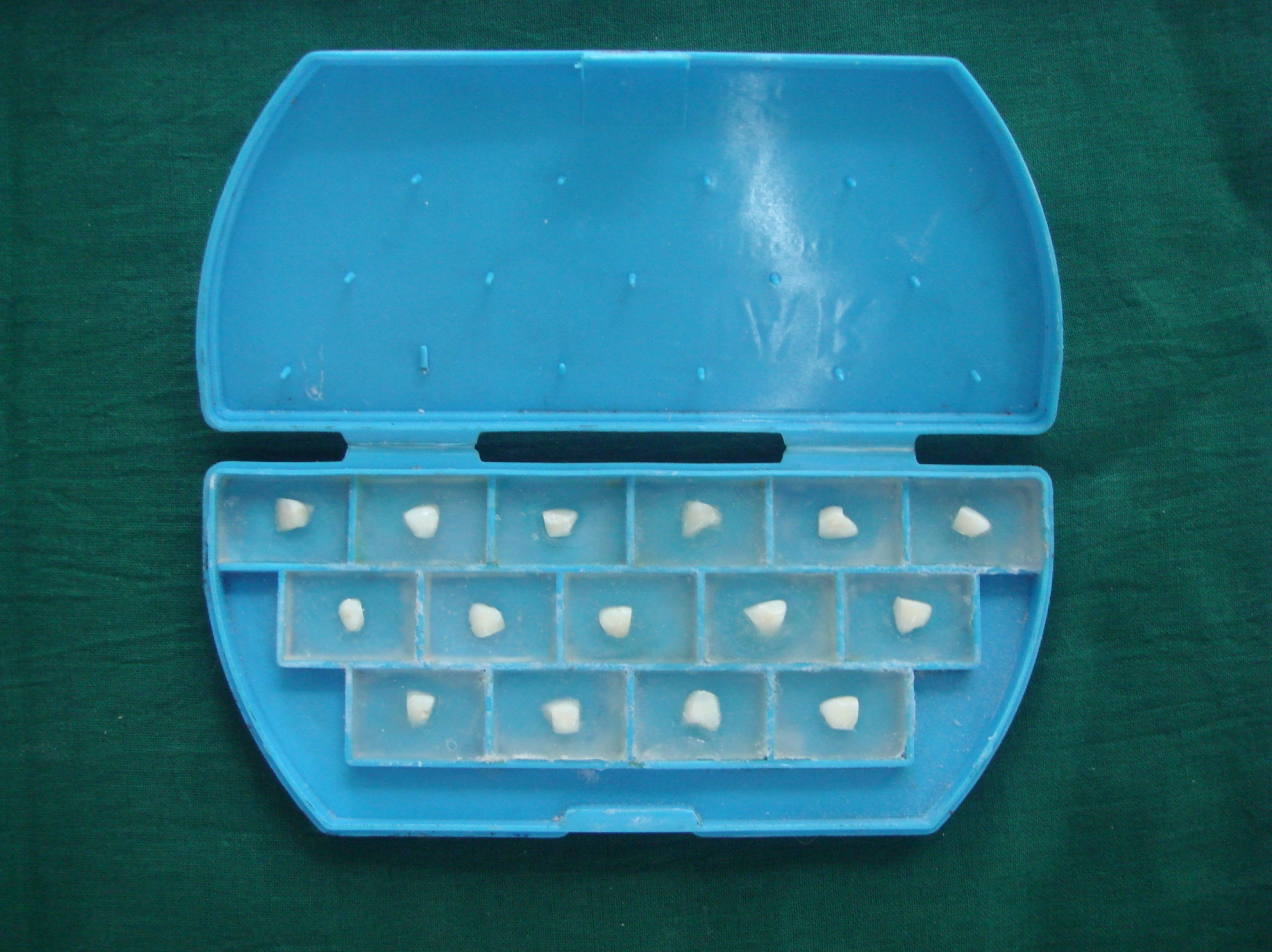
Mounting of the sections in self-cure acrylic resin

Sections were mounted individually in self-cure acrylic resin with the enamel surface facing the top (Figure 2). 

**Figure 3:**
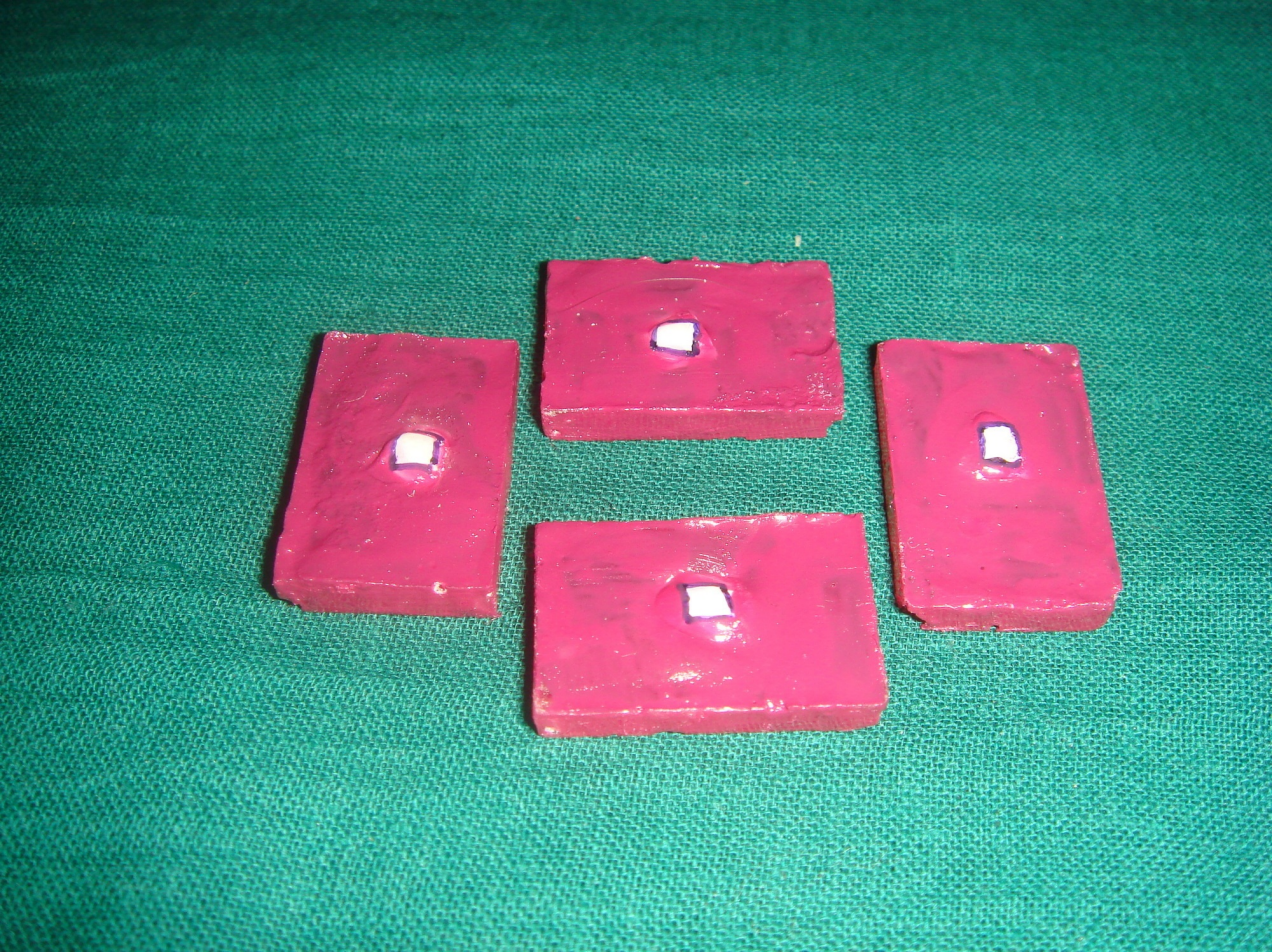
Sample after the application of nail varnish

A 3 mm x 3 mm treatment window was then opened on each tooth section by applying acid-resistant nail varnish (Revlon, New York, USA) [7] on all other areas of the acrylic block except for the area designated as the treatment window. They were then labeled according to the treatment protocol (Figure 3).

Lesion formation

The specimens were kept in the demineralization solution  (2.2 mM CaCl_2_ 2.2 mM KH_2_PO_4_, 0.05 M lactic acid, 0.5 ppm fluoride; pH adjusted to 4.5 using 50% NaOH solution) for 72 hours, at 37°C. After 72 hours of demineralization, the specimens were washed thoroughly in distilled water to remove any traces of the solution [8].

pH-cycling treatment protocol

**Figure 4:**
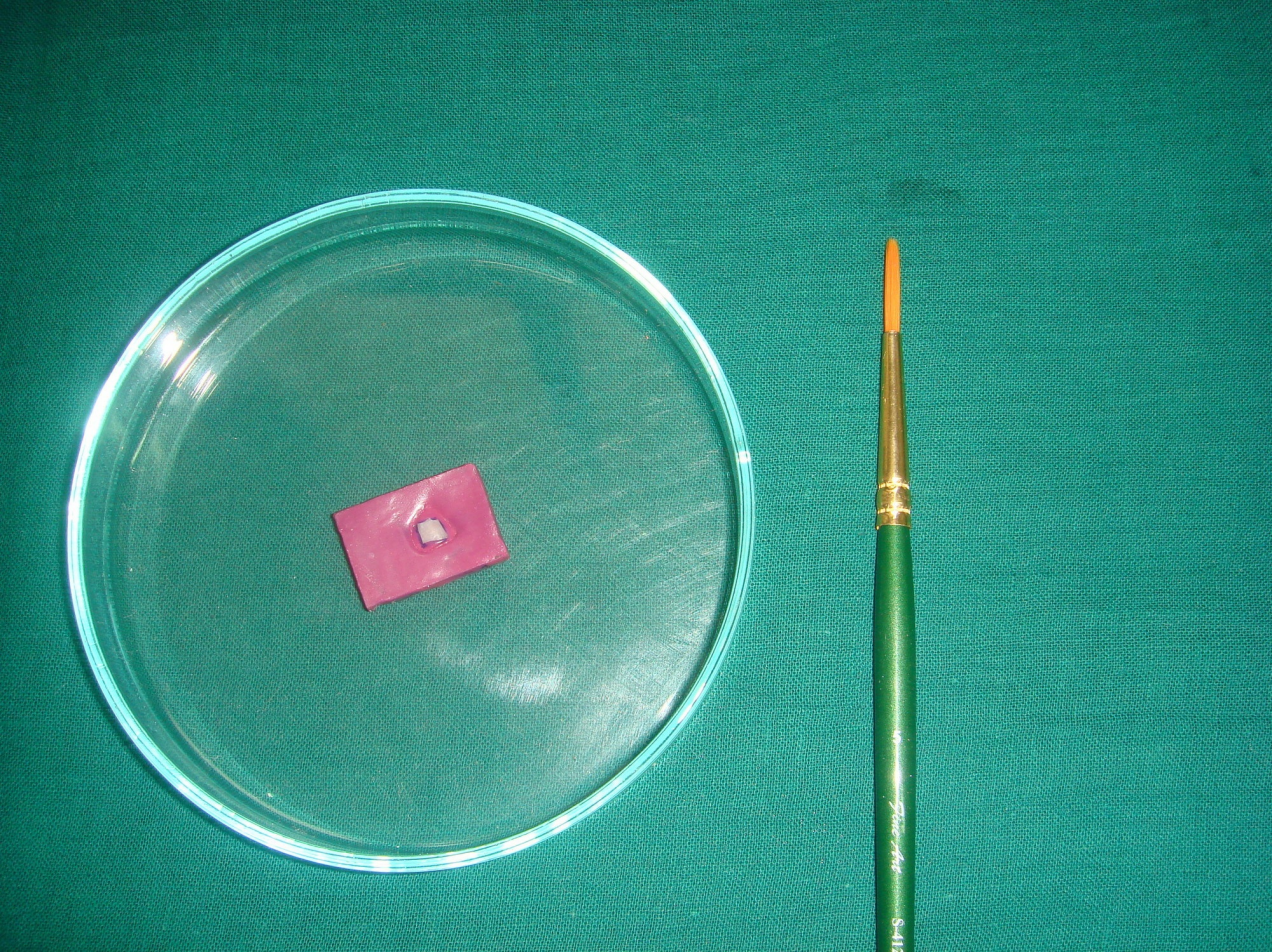
Group A: no treatment

**Figure 5:**
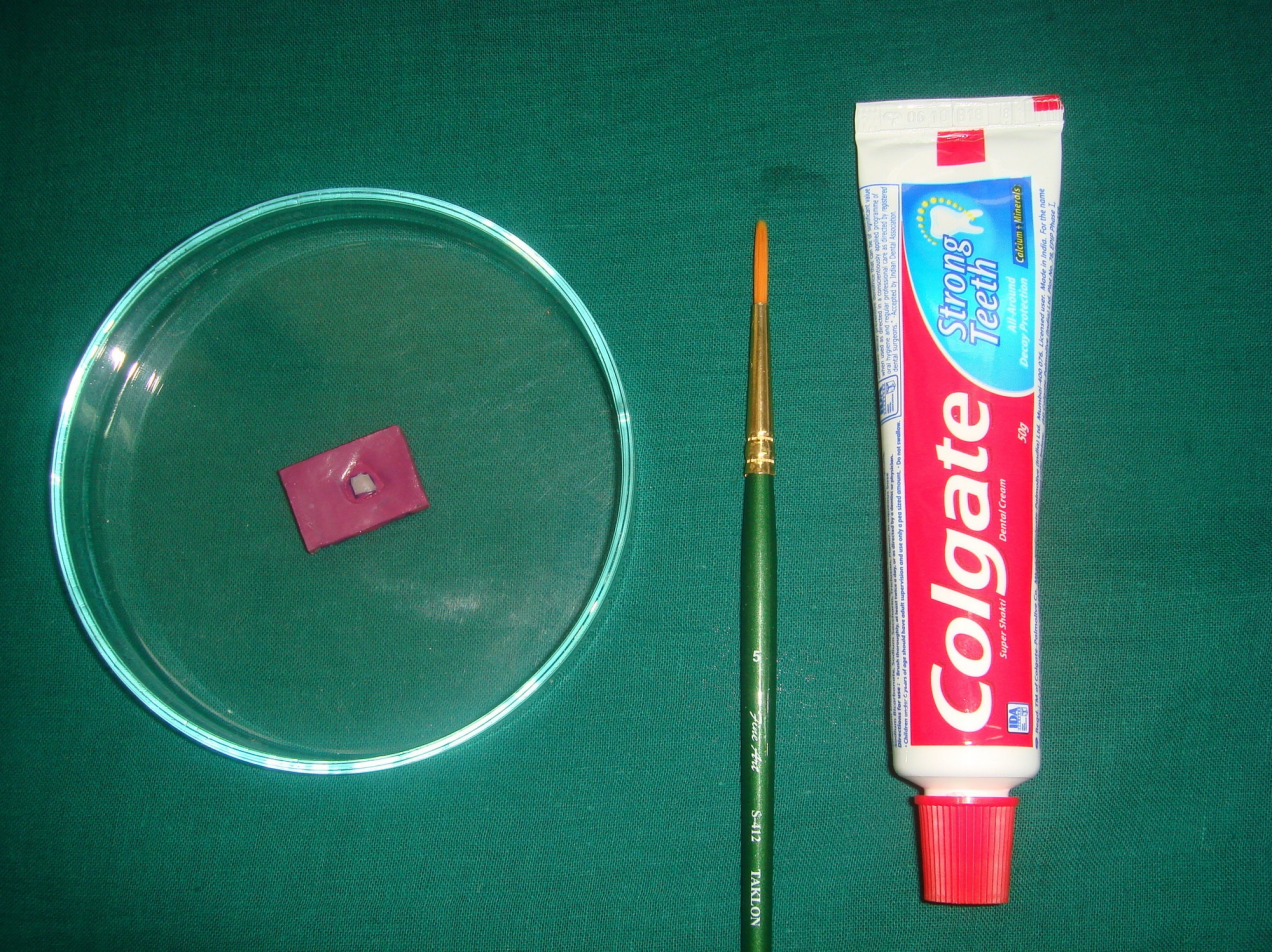
Group B: monofluorophosphate treatment

The tooth specimens were subjected to a pH-cycling protocol, including alternate immersion in the demineralization solution (detailed above) and the remineralization solution (3.90 mM Na_3_PO_4_, 17.98 mM KCl, 0.08 mM MgCl_2_, 3.27 mM NaHCO_3_, 4.29 mM NaCl, 1.10 mM CaCl_2_ and 0.50 mM H_2_SO_4_; pH adjusted to 7.2 using 85% lactic acid at 37ºC with in-between dentifrice treatment done at room temperature [9]. The dentifrices used were monofluorophosphate dentifrice (Colgate Regular; Colgate-Palmolive (India) Ltd.) containing 1000 ppm fluoride, casein phosphopeptide-amorphous calcium phosphate (GC Tooth Mousse, GC Corporation, Tokyo, Japan) containing 10% w/w CPP-ACP and calcium sodium phosphosilicate dentifrice (SHY NM, Group Pharmaceuticals Ltd., Mumbai, India) containing 5% w/w Novamin for a period of five days. The first quadrant was not given any dentifrice treatment and was designated as group A (Figure 4),

The second quadrant was treated with monofluorophosphate dentifrice and designated as group B (Figure 5).

**Figure 6:**
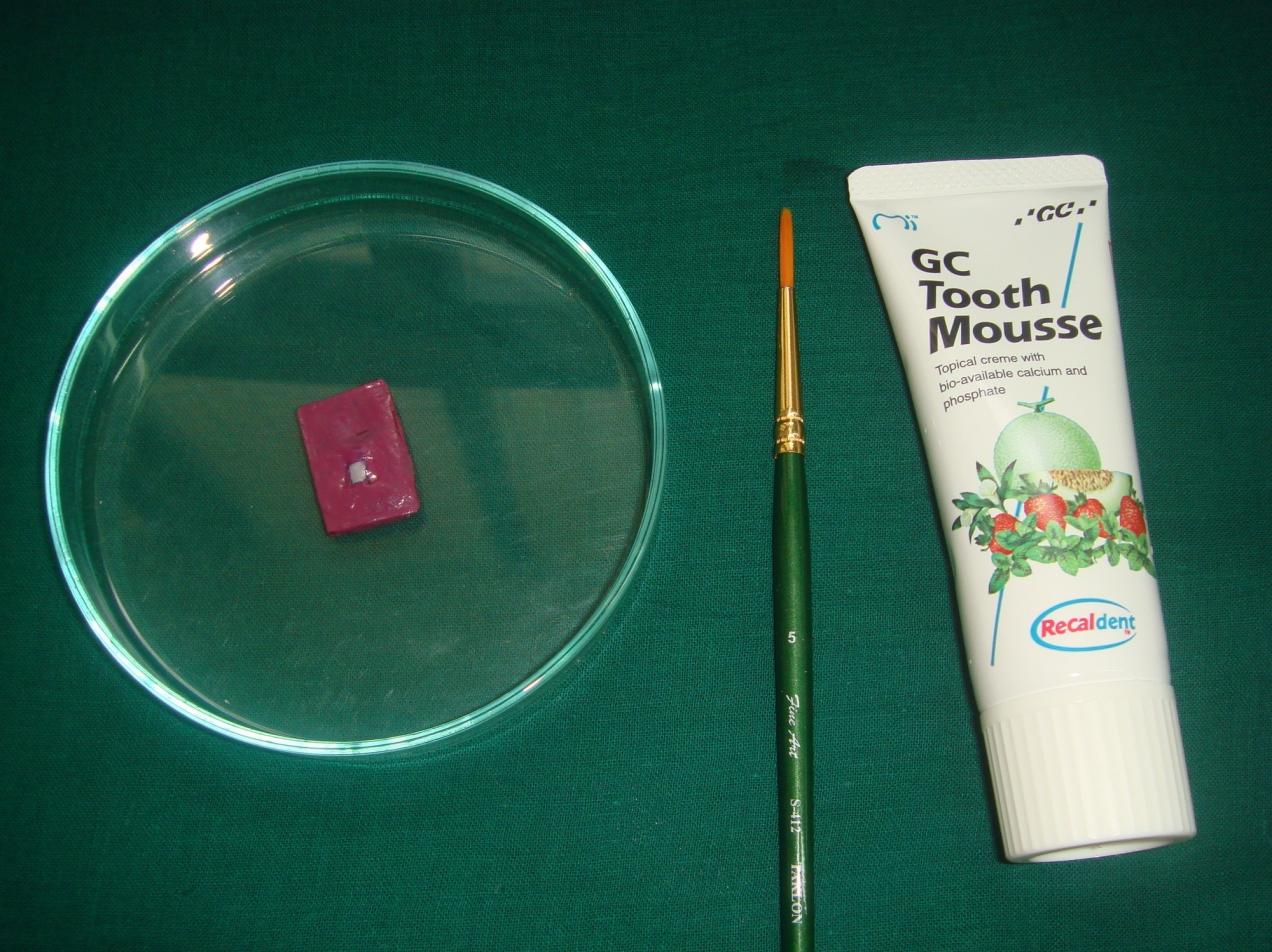
Group C: CPP-ACP treatment CPP-ACP: casein phosphopeptide-amorphous calcium phosphate

The third quadrant was treated with CPP-ACP and was designated as group C (Figure 6).

**Figure 7:**
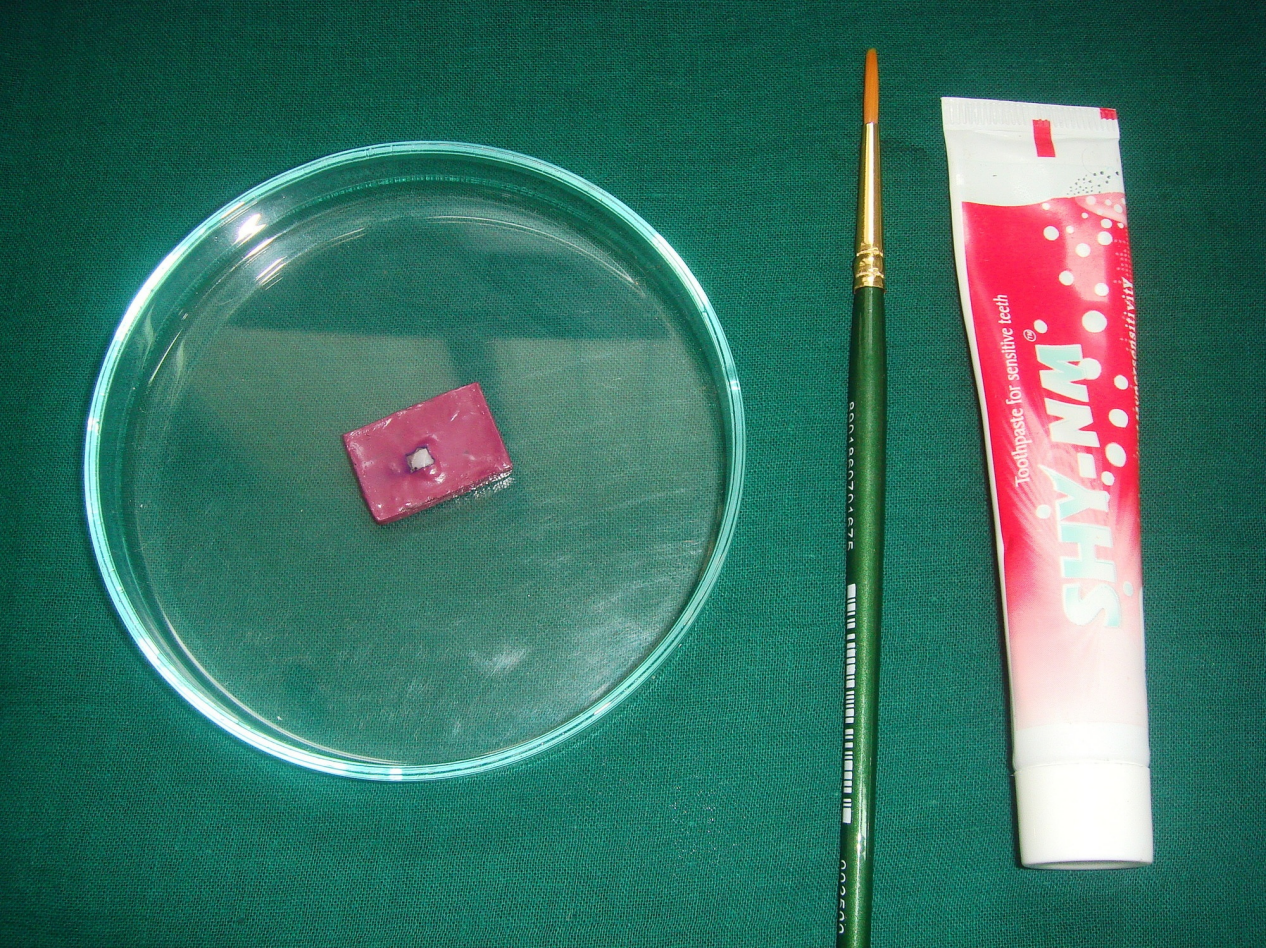
Group D: calcium sodium phosphosilicate (Novamin) treatment

The fourth quadrant was treated with calcium sodium phosphosilicate and was designated as group D (Figure 7).

The following protocol was followed [10]:

1)  Applying the test materials on the specimens

2)  Placing the specimens in the demineralizing solution for three hours

3)  Washing the specimens in distilled water

4)  Applying the test materials on the specimen

5)  Washing the specimens in distilled water

6)  Placing the specimens in synthetic saliva for 20 hours

Cross-sectioning and staining

**Figure 8:**
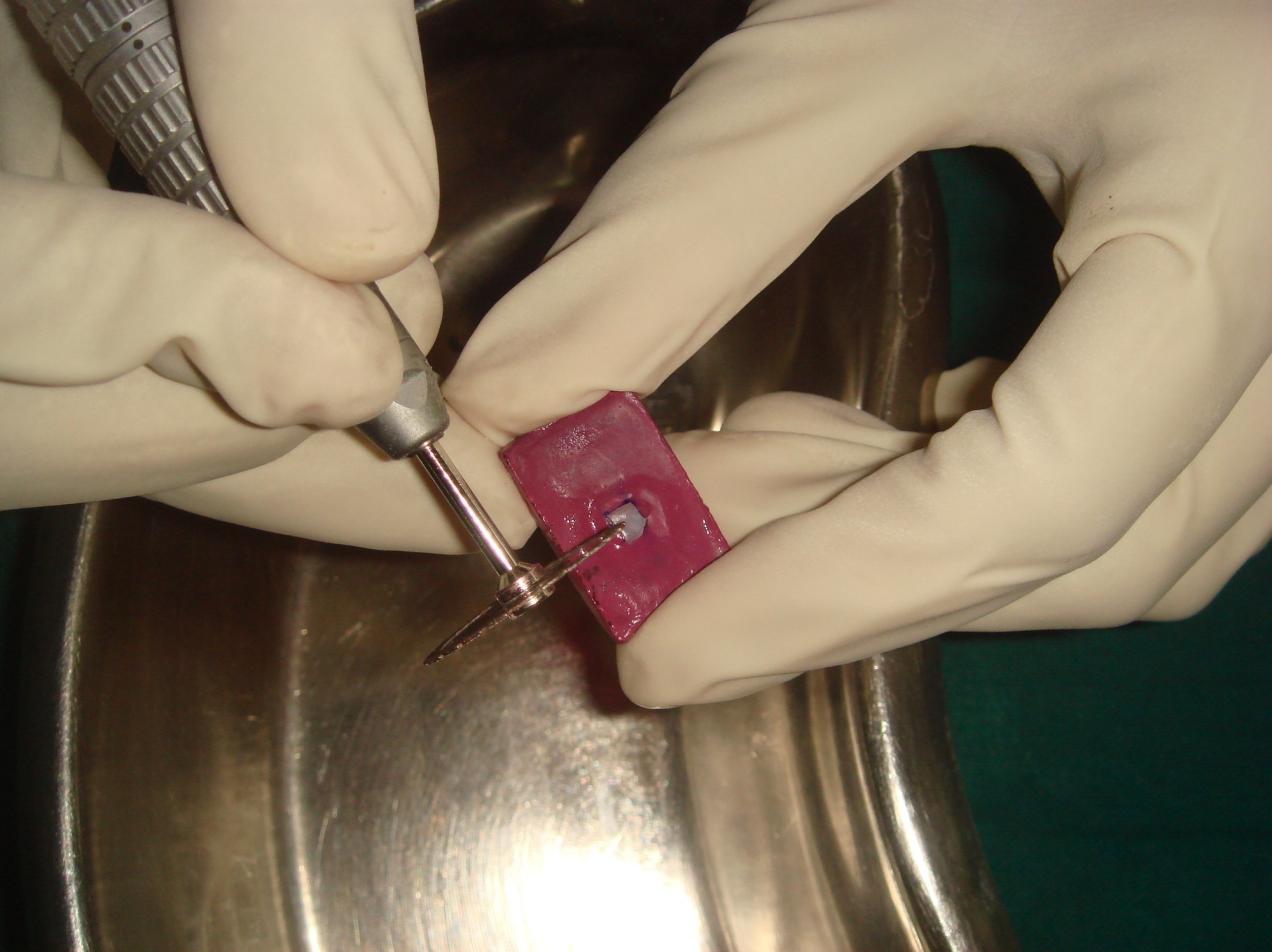
Cross-sectioning through the midline

The specimens were sectioned across the treatment window to expose the lesion depth (Figure 8).

 Each section was stained by immersion in 0.1 mM rhodamine solution for 24 hours [8].

Confocal laser microscopic analysis

**Figure 9:**
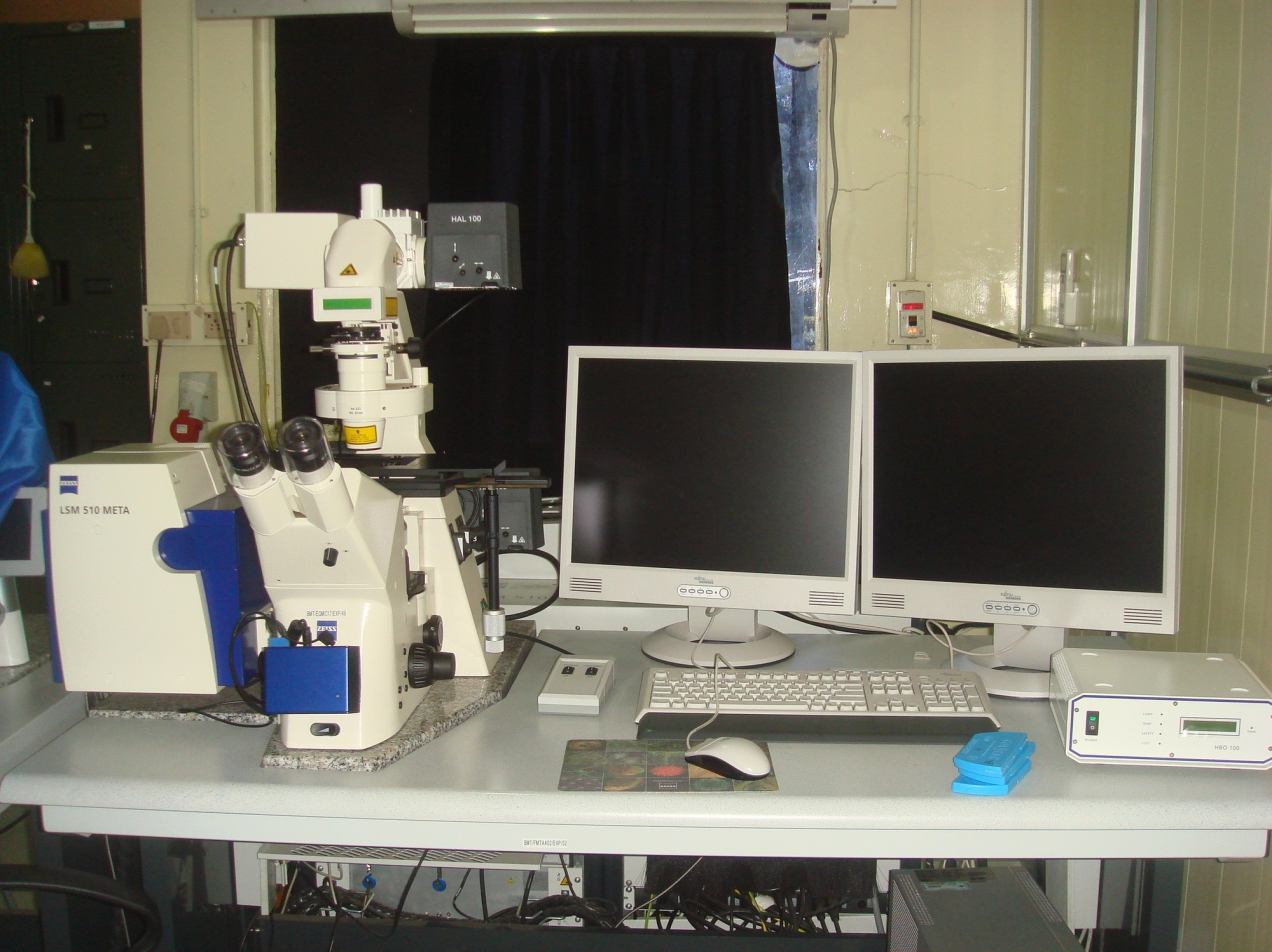
Confocal laser scanning microscope

The sections were subjected to a cross-sectional confocal laser microscopic analysis at the Department of Experimental Pathology, Sri Chitra Thirunal Institute of Medical Sciences. A Carl Zeiss Axiovert 200M inverted microscope (Göttingen, Germany) with a confocal attachment was used for this study (Figure 9).

**Figure 10:**
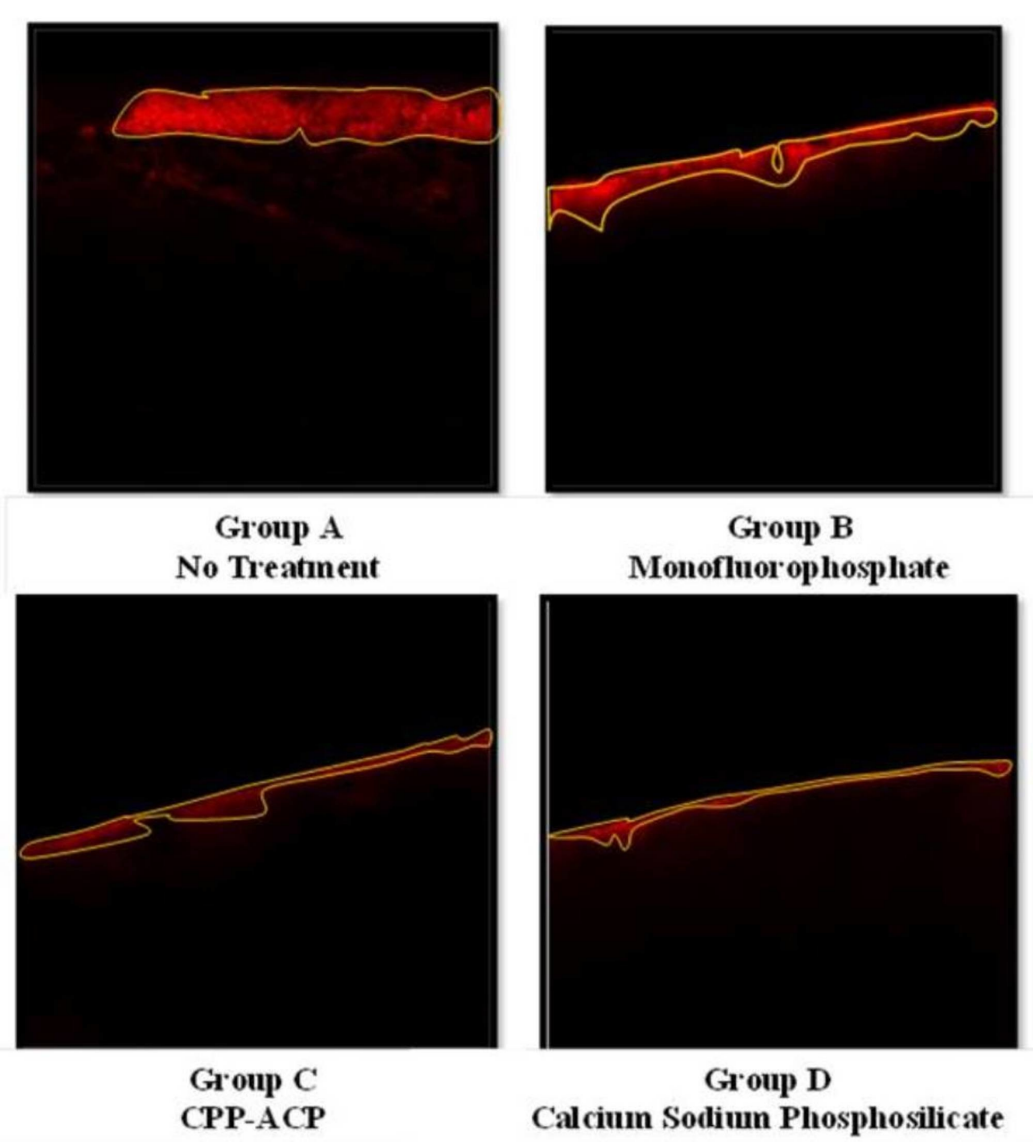
Representative images

This microscope used Carl Zeiss LSM 510 version 4 software (Göttingen, Germany) in order to visualize the images captured by the microscope on a computer screen. The cross-sectional analysis was based on digital images taken at controlled microscope settings. The area stained by the rhodamine dye appears red and is indicative of a demineralized area. Sound tooth and acrylic resin appear black, as they do not absorb any dye. Representative images are shown (Figure 10).

Image analysis

Image analysis was done using the imageJ software (National Institutes of Health, Maryland, US) and the following parameters were analyzed.

1. Cross-sectional lesion area

2.Total fluorescence (total gray value)

## Results

The mean area and mean total fluorescence for the no treatment group were recorded as 16449.2 µ2 and 759743.1, respectively. The group treated with monofluorophosphate dentifrice recorded the values as 73712 µ2 and 233765.9. The values for the lesion area and total fluorescence for casein phosphopeptide-amorphous calcium phosphate group were 5776.6 µ2 and 129470.8 respectively. The lowest values for lesion area and total fluorescence were recorded by the calcium sodium phosphosilicate group as 3874.1 µ2 and  107282.6, respectively. One-way ANOVA showed that the results were statistically significant (p<0.01) (Tables 1-2).

**Table 1:** Comparison of cross-sectional demineralized lesion area based on group Actual P value is <2e-^16 ^

	Mean	SD	N	F	P value
Group A No treatment	16449.2	247.2	30	10269.06**	0.000
Group B Monofluorophosphate	7371.3	113.1	30		
Group C Casein Phosphopeptide-Amorphous Calcium Phosphate	5776.6	264.5	30		
Group D Calcium Sodium Phosphosilicate	3874.2	468.2	30		

**Table 2:** Comparison of total fluorescence (total gray value based on group Actual P value is <2e-16

		Mean	SD	N	F	P value	
Group A No treatment		759743.1	4847.4	30	81616.44**	0.000	
Group B Monofluorophosphate		233765.9	2168.6	30			
Group C Casein Phosphopeptide-Amorphous Calcium Phosphate		129470.8	4261.2	30			
Group D Calcium sodium phosphosilicate		107282.6	9577.2	30			

Scheffe multiple comparisons proved that the variations in each group were also significant. As the lowest values were recorded by the calcium sodium phosphosilicate group, it emerged as the most effective remineralizing agent in this study. It was followed by CPP-ACP. Monofluorophosphate was proved as the least effective remineralizing agent as the values recorded for lesion area and total fluorescence were the highest for this group.

## Discussion

Fluoride is the most commonly used remineralizing agent. By acting on the dynamics of the caries process, fluoride is very effective in slowing down the progress of carious lesions. It not only inhibits demineralization but also aids in the remineralization of the enamel surface, leading to the formation of fluorohydroxyapatite or fluorapatite.

A potential problem with the mechanism of fluoride in remineralization is that for fluorapatite or fluorohydroxyapatite to form, calcium and phosphate ions are required, as well as fluoride ions. Several authors have now shown that enamel remineralization in situ and the retention of fluoride in plaque are dependent on the availability of calcium ions [11-12].

CPP-ACP and calcium sodium phosphosilicate are both based on calcium and phosphate ions. Calcium on the surfaces of the calcium and phosphate ion clusters primarily interacts with the casein phosphopeptides through the negatively charged residues of the peptides [3]. Casein phosphopeptides only weakly bind calcium and phosphate ions, thus allowing for a dynamic equilibrium between free and bound ions. This, therefore, provides a reservoir of bioavailable ions, which, on changes in the intraoral pH, can be easily released to aid in the process of remineralization.

In aqueous environments, Na+ particles in calcium sodium phosphosilicate immediately (within one minute) begin to exchange with hydrogen cations (H+ or H3O+) [13-15]. This rapid exchange of ions allows calcium (Ca2+) and phosphate (PO43–) species to be released from the particle structure. A modest, localized, transient increase in pH occurs that facilitates the precipitation of calcium and phosphate from the particles and from saliva to form a calcium phosphate (Ca-P) layer on tooth surfaces. As the reactions and the deposition of Ca-P complexes continue, this layer crystallizes into hydroxycarbonate apatite, which is chemically and structurally similar to biological apatite [13]. The results obtained showed that samples treated with calcium sodium phosphosilicate recorded the least values for the demineralized cross-sectional lesion area and total fluorescence. This suggests that very few demineralized areas were present in these samples. As the dye stains only demineralized areas, the area occupied by the dye was the least in these specimens, consequently, giving rise to the lowest values for the parameters measured. The values recorded for cross-sectional demineralized lesion area and total fluorescence were highest for the no treatment group, as they were not subjected to any surface treatment. This reinforces the fact that some form of dentifrice usage is a must for remineralization to occur and saliva alone is not adequate. The values recorded by the CPP-ACP group were higher than the calcium sodium phosphosilicate group, suggesting that the remineralization produced by the latter was more effective. Monofluorophosphate lead to minimal remineralization in this study.

Though casein phosphopeptide-amorphous calcium phosphate aids in subsurface remineralization, its use as-an-over the counter dentifrice has not been promoted. The cost of such products still remains high, and there is a need for their wider availability leading to increased usage and the benefits of the casein phosphopeptide-amorphous calcium phosphate technology. Another matter of concern is that plaque peptidases and phosphatases can degrade the phosphopeptides. The dephosphorylation of the phosphoserines of the Casein phosphopeptides by phosphatases substantially reduces the ability of the peptides to bind calcium and phosphate ions [16]. This leads to a loss of valuable calcium and phosphate ions and thus would hamper remineralization.

Calcium sodium phosphosilicate comes as a more economical alternative to CPP-ACP and according to this study, it is more effective than CPP-ACP. However, one consideration that must be kept in mind is that the remineralization with calcium sodium phosphosilicate leads to the formation of carbonate apatite while that with fluoride leads to the formation of fluorapatite. Fluorapatite is more resistant to further acid attack than carbonate apatite. The use of fluoride dentifrice can, therefore, be supplemented with calcium sodium phosphosilicate dentifrice for achieving better remineralization, as fluoride dentifrice is the most common and most economical dentifrice used in most households in India. Hence, measures to use a combination of calcium sodium phosphosilicate and fluoride must be explored.

This study was an in vitro study. In vitro models are mechanistically limited in three key ways: an inadequate simulation of the biological aspects of caries, a difficulty in matching solid/solution ratios occurring in vivo, and artifacts associated with substrate choice/reaction conditions

To obtain meaningful data, in vitro analysis requires the investigator to eliminate as much experimental variability as possible. The relative success of this study should be credited to meticulous standardization in each procedural step. Sample preparations, solutions, and processes were all subject to strict uniformity control for all tooth sections. This leaves only inherent specimen variability unaccounted.

Therefore, within the limits of the study, the results of using calcium sodium phosphosilicate as a remineralizing agent seem promising. Further clinical trials are, however, necessary to make public recommendations.

## Conclusions

The results of this study suggest that among the three agents tested, calcium sodium phosphosilicate is the most effective remineralizing agent followed by casein phosphopeptide-amorphous calcium phosphate. Though monofluorophosphate is effective as a remineralizing agent, its effectiveness is not as good as the other two agents when tested under the conditions mentioned in this study.
